# Nerves and Epidemics

**Published:** 1890-01-11

**Authors:** 


### THE DOCTOR'S PULPIT.

### NERVES AND EPIDEMICS.

The epidemic is fairly upon us at last. The daily newspapers have for some time been resolved that we should have it, and now it has come. They are determined, too, that the cause of the outbreak is a specific microbe. Happy newspapers! No "honest doubts" disturb the serenity of their scientific assurance. For our part, microbe or no microbe, we believe in prevention; and where prevention is not secured, in practical modification by means of the will.

There is no better established fact in the whole history of epidemics than this, that the man or the woman of pluck and energy is the last to take the prevalent disease, and the surest to get safely through it if he does take it. The power of mind and will over the physical organism is immense. This is illustrated in a thousand ways: on the battle-field; in the desert; in the malarious African swamps; at sea; in fever dens at home, and in a hundred other places. It is universally believed, both by medical men and the public, that fear is one of the most potent factors in every dangerous epidemic. It is easy to understand why this should be so. Fear worries the nervous system. But whatever worries the nervous system diminishes the appetite, impairs the digestion, and disturbs the sleep. All these conditions are injurious to health, and in the same proportion conducive to the acquirement of external diseased poisons.

It may be supposed by some that if the present influenza epidemic is caused by a specific microbe, and by that alone, there is no hope of resisting the infection if we come in contact with the microbe. But this comes of a total misapprehension of scientific facts. There are certain conditions of the system in which a single glass of sherry will intoxicate a man. In other conditions, the same man might take six glasses of sherry without being the least intoxicated. We are not, of course, saying that sherry has any kinship with a living microbe. We are merely pointing out the very varying powers of resistance that may be stored up in the human body at different times. It is just as certain that as a man in full health, and with a stomach satisfied with food, can resist the intoxicating effect of sherry much more decisively than a weak man with an empty stomach, so can a man of energetic health and resolute nerves resist the multiplying power of a dangerous microbe much better than his weaker-bodied and feebler-willed fellow man.

The first thing, then, for men and women to do is to keep their minds easy, and as far as possible to forget that there is an epidemic in existence. Ladies and weak-nerved men will do well to avoid the influenza column of the daily paper. It is no part of the business of the writers of daily newspaper articles to consider the health of their readers. Their first duty is, of course, to supply information, and to make their wares sell. But if the daily papers could be "gagged" for a fortnight, a proceeding which we do not suggest, the epidemic would probably be over. Next in importance to an easy mind is regularity and full enjoyment in eating. We have already said that fear impairs the appetite. To counteract this impairment, all persons would be well advised to spend a little additional money in making all their meals more than usually appetising. Not only so, but they should make up their minds to sit longer at table, so as to give digestion a better chance.

The present writer sees very considerable danger for those who live at considerable distances from home and have to travel by train to business every day. There are always a large number of selfish and wrong-headed persons who insist upon having the windows of railway carriages open whatever the state of the weather may be. An east wind, a driving rain, a snowstorm—no matter what—it is all the same to them. They are so intent upon breathing pure air that they would rather die with well oxidised blood than live with an infinitesimal quantity of carbonic acid in excess. They may be right. But so far as the other passengers are concerned it would be better if they did die than that they should give every man in the compartment rheumatism, asthma, bronchitis, or the "grippe." For the next two or three weeks, at any rate, the majority of railway passengers should insist upon having the windows closed to the top whilst the train is in motion. They may, of course, be opened for a few minutes at each stopping-place. There is really no great harm in sitting for half an hour in a railway carriage with the windows shut. The air of the compartment is warmer and more rarefied than the outside air, and as a consequence the fresh air is con stantly rushing in at every available chink and crack. Whoever doubts this has only to sit with his rheumatic shoulder close to the glass of the compartment, and a quarter of an hour will be more than sufficient to convince him of his error.

It is surprising how little discretion many people exercise about their clothing during the winter season. If they begin with thick woollen garments in November they continue with thick woollen garments until March. It does not matter though the temperature be 25° one day and 55° the next, the same garments must be worn each day. They complain that if they throw off their overcoats on the warmer day they are sure to take cold. Quite true! But is there no alternative between wearing a thick overcoat and no overcoat at all? Of course they should not throw off overcoats entirely, neither should they wear the thin coats suitable for summer use. Everybody who can afford it should have a thin as well as a thick woollen overcoat for winter; and thin as well as thick underclothing. The thing to be borne in mind is that winter underclothing of all kinds should consist of woollen; and the next thing is that all those parts of the body that are usually covered in winter should never be uncovered, however mild the day. Men may and ought to be more lightly clothed on the warmer days, but they should never be immediately exposed to the damp chills of the wintry air.

To bring this little sermon to a conclusion, we may say that the things most requisite to secure us against the epidemic, or to carry us safely through it, are pluck, preparation, and prudence. There need be no difficulty in "screwing our courage to the sticking place," because after all there is no serious danger to persons of ordinary strength. But still, prudence and preparedness ought certainly to be exercised; and, as a matter of financial and vital economy, it is well worth while to make the utmost use of both. We cannot but repeat our conviction, for the comfort of our readers, that much more has been made of the epidemic in every way than the occasion demanded.

There is really no advantage in an attack of this kind of influenza. It does not protect from other attacks of the same disease. On the contrary, he who has once had it is more susceptible than before. Moreover, there is undoubtedly a certain amount of risk about it. Some persons will die who would not die at present but for the disease. As a mere matter of business calculation, therefore, it is well to consider the subject thoroughly, and that each family and individual should carry out an effective "plan of campaign."

